# Thermodynamic and Kinetic Characteristics of Combustion of Discrete Polymethyl Methacrylate Plates with Different Spacings in Concave Building Facades

**DOI:** 10.3390/polym13010167

**Published:** 2021-01-05

**Authors:** Weiguang An, Lujun Peng, Minglun Cai, Kaiyang Hu, Song Li, Tao Wang

**Affiliations:** 1 Jiangsu Key Laboratory of Fire Safety in Urban Underground Space, China University of Mining and Technology, Xuzhou 221116, China; TS19120027A31@cumt.edu.cn (L.P.); TS19120073P31@cumt.edu.cn (K.H.); TS19120014A31TM@cumt.edu.cn (S.L.); TS19120031A31@cumt.edu.cn (T.W.); 2 Key Laboratory of Gas and Fire Control for Coal Mines, China University of Mining and Technology, Ministry of Education, Xuzhou 221116, China; 3 State Key Laboratory of Coal Resources and Safe Mining, China University of Mining and Technology, No. 1 University Road, Xuzhou 221116, China

**Keywords:** polymethyl methacrylate, thermodynamics and kinetics, combustion, discrete flame spread, concave building facade

## Abstract

Polymethyl methacrylate plates are widely applied to buildings, producing significant fire hazards. It lacks a theoretical basis for the fire risk assessment of polymethyl methacrylate in concave building facades. Therefore, experimental methods are used to investigate combustion characteristics of discrete polymethyl methacrylate plates in a concave building facade. Influences of fuel coverage and structure factor are investigated, which is scant in previous works. When structure factor is invariable, average flame height increases first and then decreases as fuel coverage increases, and the turning point is between 0.64 and 0.76. In total, three different patterns of pyrolysis front propagation are first observed for different fuel coverages. Flame spread rate first increases and then decreases as fuel coverage rises, and the turning point is also between 0.64 and 0.76. When fuel coverage is invariable, the flame spread rate first increases and then decreases with increasing structure factor, and the turning point is 1.2. A model for predicting the flame spread rate of discrete polymethyl methacrylate is also developed. The predicted values are consistent with experimental results. Fuel spread rate of discrete polymethyl methacrylate rises as the fuel coverage increases. The above results are beneficial for thermal hazard evaluation and fire safety design of polymethyl methacrylate used in buildings.

## 1. Introduction

**Figure 1:**
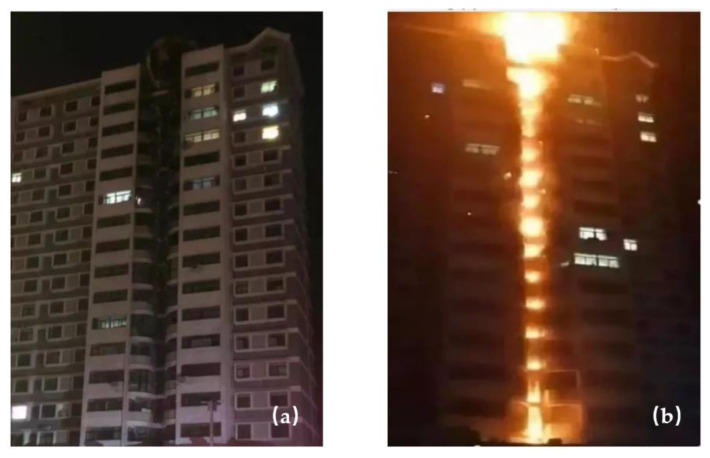
(**a**) Concave structure of a building; (**b**) a fire occurring in the concave structure.

Polymethyl methacrylate (PMMA) is widely used as a building roof and a curtain wall for natural lighting, which can reduce building energy consumption by 33–78% [1,2]. Moreover, the thermal conductivity of PMMA is much lower than that of glass, leading to the better thermal insulation properties. In addition, the chemical stability, mechanical properties and weather resistance of PMMA are outstanding [3]. Therefore, PMMA is a relatively important material in building thermal engineering. However, PMMA without flame retardant is flammable, releasing a lot of toxic gas and resulting in rapid flame spread, which enhances building fire hazards [4,5]. The fire hazard of PMMA is significantly affected with distribution of materials and structures of building facade. Under most conditions, the distribution of PMMA is discrete, rather than continuous (Figure 1a) [6]. As a common building structure, the concave facade obviously influences combustion behaviors of building materials [7]. For example, in March 2019, a fire occurred in Kaifeng, China (Figure 1b) [8]. Under the influence of the concave building facade, it took only 2 min for the fire to spread to the top of the building from the bottom. Therefore, it is necessary to investigate combustion behaviors of discrete PMMA plates with different spacings in concave building facade.

Concerning different building structures, such as concave facade, ceiling inclination, curtain wall channel [9], corner of wall, etc., some works have been conducted to investigate their influences on combustion behaviors. Moreover, studies concerning flame spread over PMMA plate are also reviewed in this section. A heat transfer model was proposed by Zhu et al. [4,5] to predict the downward flame spread rate of PMMA in a curtain wall channel with different spacings. Moreover, as the spacing increased, the flame height, preheating zone length and mass loss rate all increased first and then decreased. Matsuoka et al. [10] investigated the geometrical effects on the flame spread over thermally thick combustibles in a narrow channel. They found that the flame spread rate increased with channel height when the channel height was sufficiently small, and then the flame spread rate started to decrease as the channel height further increased. Zhou [11] conducted an experimental study on average fire propagation speed (FPS) of the external wall using the expanded polystyrene thermal insulation system, finding that the relationship between the average FPS and the vertical distance is positively linear. Tao [12] studied the ignition behavior of different shapes of PMMA exposed to external heat radiation and found that cylindrical samples are easier to ignite than flat samples. The ignition time dropped with the decrease of the sample radius. Peng et al. [13] found the flame spread rate of PMMA presented a “U” variation with the increasing of ceiling inclination. They proposed a correlation among pyrolysis length, sample width and ceiling inclination. Gao et al. [14] found the flame spread rate of PMMA is proportional to flame width under the influence of curtain wall channel. The flame height increased linearly with time. Cai et al. [9] studied the downward flame spread over thermal insulation materials in an enclosed vertical channel, and found that the total heat flow received by the preheating zone is mainly controlled by flame convection. Chu et al. [15] studied the upward flame spread of cotton fabrics with different moisture regain, and observed that the flame height and flame spread rate kept stable first and then decreased with an increase in the moisture. Liang et al. [16] revealed the sidewall constraint effects of a channel on combustion characteristics of the fire source and the temperature distribution. Zeinali et al. experimentally studied the fire characteristics and flame spread behaviors in a corner of building walls [17,18]. Jiang et al. [19] studied the fire safety of thermal insulation materials over a wide range of widths and established the heat transfer model for flame spread over thermal insulation materials. Ma et al. [20] studied the influence of facade structures on parallel, symmetric and adjacent buildings on downward flame spread characteristics of polyurethane (PUR)and found that the flame spread rate and flame height increased first and then decreased as the angle of the adjacent facade increased and reached their maximum when the critical angle was 90°; Tao et al. [21] revealed the influence mechanism of the spacing between two side walls of concave channel on buoyant jet flames, built the prediction model of virtual ignition source, and found that the flame height increases as the spacing between side walls decreases. Gao et al. [22] conducted a set of burning experiments to investigate the restriction influence of sidewalls and established empirical equation to predict the maximum gas temperature. Tsai [23] studied the influence of sidewalls on the width effect of vertical flame spread and found that the sidewalls increase the flame height and lower the heat feedback at flame center. Yan et al. [7,24] conducted an experiment concerning upward and downward flame spread behaviors at different altitudes and found that under higher pressure, the upward flame spread rate over concave facade is higher, and both flame spread rate and mass loss rate tend to rise as the structure factor increases, revealing the mechanism of how the concave facade influences the vertical flame spread. On the basis of above works, An et al. [25] further discussed the influence of concave facade on the upward flame spread over thermal insulation materials, deduced the formula concerning between dimensionless flame height and structure factor, and built the model to predict flame spread characteristics under the influence of the concave facade.

Most previous works focused on combustion behaviors of continuous materials. However, studies concerning the combustion of discrete combustibles were relatively less. Park and Liao [26] studied the influence of air gap on the vertical flame spread of thermally thin materials through numerical simulation and small-scale experiments, finding two aspects of the influence: one is the jumping phenomenon at the flame bottom and flame front, and the other is that the air gap makes upward flames closer to the fuel surface, leading to stronger flame heat flow received by the fuel surface. Cui et al. [27] further studied the influence of air gaps with different lengths on the vertical flame spread over thin filter papers, and found that as the air gap gets larger, the flame spread rate and mass loss rate increases first and then decreases. Miller et al. [6] studied the upward flame spread characteristics of PMMA when noncombustible isolation strips are arranged at the same spacing, and indicated that the flame spread rate increases first and then decreases as fuel coverage (ƒ) decreases, and reaches its maximum when ƒ = 0.64. Wang et al. [28] indicated that the average flame spread rate increases as fuel coverage rate rises. In addition, there is a positive correlation between average flame height and fuel coverage. More scholars [29,30,31,32] studied discrete flame spread over matchstick arrays. Jiang et al. [33] conducted a series of combustion experiments of wood plug arrays with different spacings, found that the vertical flame spread rate is significantly larger than the average flame spread rate, and predicted the average flame spread rate and mass loss rate using radiation control model. 

Beside the heat hazards, polymer fires also produce a variety of organic pollutants. Altarawneh et al. [34] found that the pyrolysis products of permethrin include dibenzo-p-dioxins (PCDD) and polychlorinated dibenzofurans (PCDF), which may cause pollution to the environment. In addition, they [35] investigated the interaction between a 2-chlorophenol molecule and Cu_2_O(110): CuO surface and they found the formation of a 2-chlorophenoxy moiety, which is one of the organic pollutants in combustion systems.

In conclusion, previous studies mainly focused on analyzing combustion characteristics of continuous materials in different building structures, ignoring the discrete distribution of materials, which is commonly observed in the real building fire scene. In fact, due to the existence of windows, corridors, fire barrier zones and various gaps, most combustible materials are distributed in a discrete form. At present, some scholars have carried out researches on discrete flame spread, but most of them only conduct qualitative analysis, without establishing relevant mathematical models or considering the influence of architectural structure. This leads to a lack of a theoretical basis for fire risk assessment and fire protection design of buildings with special external wall structures and discrete combustible materials. In this paper, the small-scale experiment was carried out to study the combustion characteristics of discrete PMMA plates in a concave building facade. Through changing concave structure factors and spacings among discrete PMMA plates, their influence mechanism on flame spread is investigated and revealed based on analyzing thermodynamics and kinetics characteristics during combustion of PMMA.

## 2. Experimental Device, Materials and Methods 

### 2.1. Experimental Device

**Figure 2:**
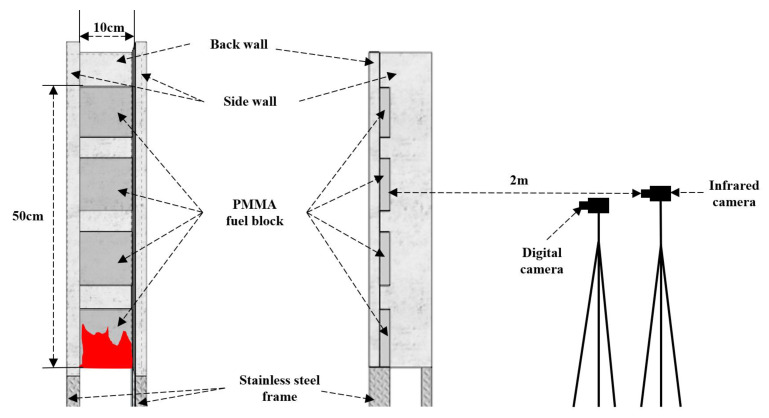
Experimental instruments.

The experimental device used in this paper is shown in Figure 2. The concave facade was made up of one back wall and two parallel sidewalls perpendicular to the back wall, which were wrapped and fixed by a frame to ensure the sealing and stability. The frame was concave, and there were four legs to support the entire concave structure. The frame was made of stainless steel since the stainless-steel frame is widely used in actual buildings to support the PMMA plates. Simultaneously, in order to effectively simulate the actual wall, non-combustible ceramic fiber boards were used on the back and sidewalls. The thermal conductivity of the ceramic fiber boards is close to the walls of real buildings. The back wall was 60 cm high and 10 cm wide. The height of side walls was 60 cm, and the width varied for different the test conditions. The ignition device is a linear pool fueled by n-heptane to ensure that sample bottom can be ignited at the same time in the experiment. 

The measuring equipment used in this experiment consisted of a digital camera and infrared camera, as shown in Figure 2. The Sony A6000 digital camera, with a data acquisition of 25 frames per second, was installed in front of the concave facade, which could record the flame shape of PMMA in real time during the whole combustion process. Then the flame height could be obtained using a further image processing method. An infrared camera (MAG30HT) with a data acquisition frequency of 25 frames per second was also placed in front of the experimental setup at a distance of 2 m, which was used to record the real-time temperature distribution of PMMA surface and its flame. The pyrolysis front of PMMA surface could also be obtained by further processing of infrared video.

### 2.2. Experimental Materials

PMMA was selected as the experimental material, whose physical properties are listed in Table 1. The thickness of the PMMA blocks was 1.5 cm, uniformly and vertically arranged on the back wall to form a continuous or discrete fuel array. PMMA is a type of thermoplastic material. Due to its stable combustion performance, PMMA is commonly used in the standard fire test. When its surface temperature reaches 160 °C, PMMA changes from a solid to a molten state. When the temperature rises to 350 °C, this material begins to pyrolysis. Multiple endothermic and exothermic processes occur during thermal decomposition of PMMA. The rapid thermal decomposition stage occurs between 350 °C and 420 °C and then it grows to be stable [5]. Its flame temperature is around 800 °C.

### 2.3. Experimental Methods

**Figure 3:**
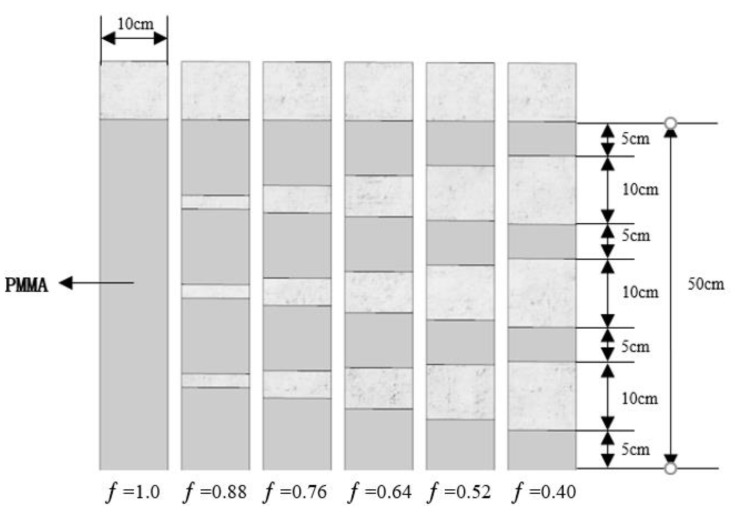
Experimental conditions concerning fuel coverage.

The experimental conditions designed in this paper included PMMA fuel coverage (ƒ) and structure factor (П). The structure factor (П) is defined as the ratio of side wall length to the back wall width. The setting of the fuel coverage and structure factor refers to a standard of China, i.e., “Code for fire protection design of buildings” (GB 50016-2014). For each test condition, the total length of PMMA fuel blocks and air gaps was fixed at 50 cm. The test conditions concerning PMMA structure factor are shown in Table 2. The distribution of PMMA fuel blocks is shown in Figure 3. The test under each condition was repeated 2 to 3 times to ensure the repeatability of the experiment and reduce experimental errors. The ambient temperature and pressure of the laboratory were 20 °C and 100.9 kPa, respectively. Since these environmental conditions changed little during the entire experiment, their effects on this study were not considered.

## 3. Results and Discussion

### 3.1. Flame Shape

**Figure 4:**
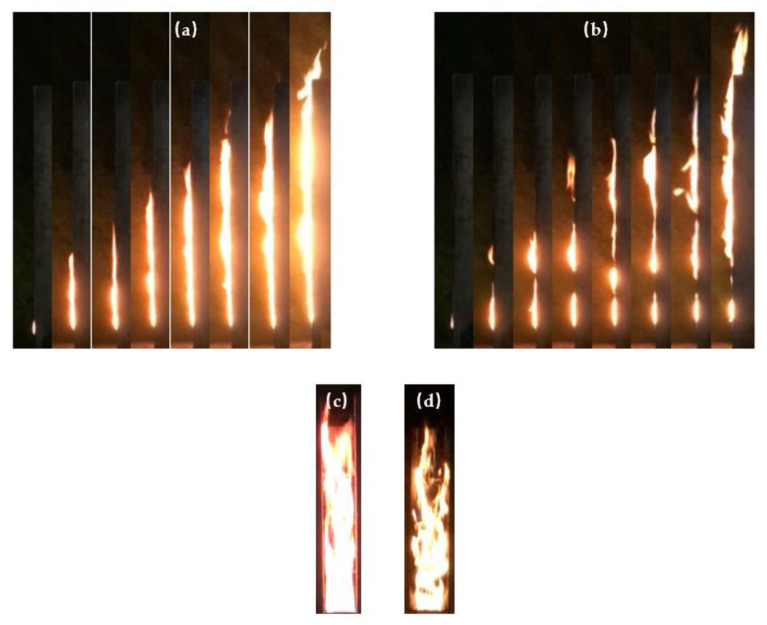
(**a**) Side view of flame shape of continuous fuels; (**b**) side view of flame shape of discrete fuels (*f* = 0.4). (**c**) Front view of flame shape of discrete fuels with sidewalls; (**d**) front view of flame shape of discrete fuels without sidewalls.

The side view of the flame shape of both the continuous and discrete PMMA flame spread without sidewalls is shown in Figure 4a,b. For the continuous PMMA flame spread, a continuous flame is formed on the surface of PMMA blocks, and both the flame thickness and shape are relatively stable. When there is an air gap between adjacent PMMA plates, the flame thickness becomes smaller, and flame “faults” are observed. The flame “faults” mean that the flame is discrete rather than continuous. The reason for the phenomena is that high-temperature pyrolysis gases flows into air gaps under the influence of front air entrainment. The air gap prevents the induced air flow rising along the PMMA surface, causing the discontinuous flame more unstable, which is similar to the phenomenon observed by Tsai [37]. When the flame reaches the top of PMMA array, it is shown in a wavy form under the influence of air gaps. The flame is thin at air gaps, while it is thick on the surface of PMMA, which is similar to the experimental phenomena in the works of Park [26] and Cui et al. [27]. The continuous flame is significantly brighter than the discrete flame. The increase of fuel coverage strengthens the heat release rate, increasing the flame brightness. Similar phenomenon was observed in Jiang et al.’s study [38].

Figure 4c,d shows the front view of the discrete PMMA flame shape. With sidewalls, the width of the PMMA front flame keeps the same as the width of PMMA material due to the restrictions of sidewalls. When there is no sidewall, high-temperature pyrolysis gas of PMMA spreads upwards and to the left and right at the same time, without any restrictions. In this case, the flame is obviously wider than the subject to side wall restrictions, and flame pulsation is more obvious, which is basically consistent with the results of An et al. [39] concerning continuous flame spread. 

### 3.2. Flame Height

**Figure 5:**
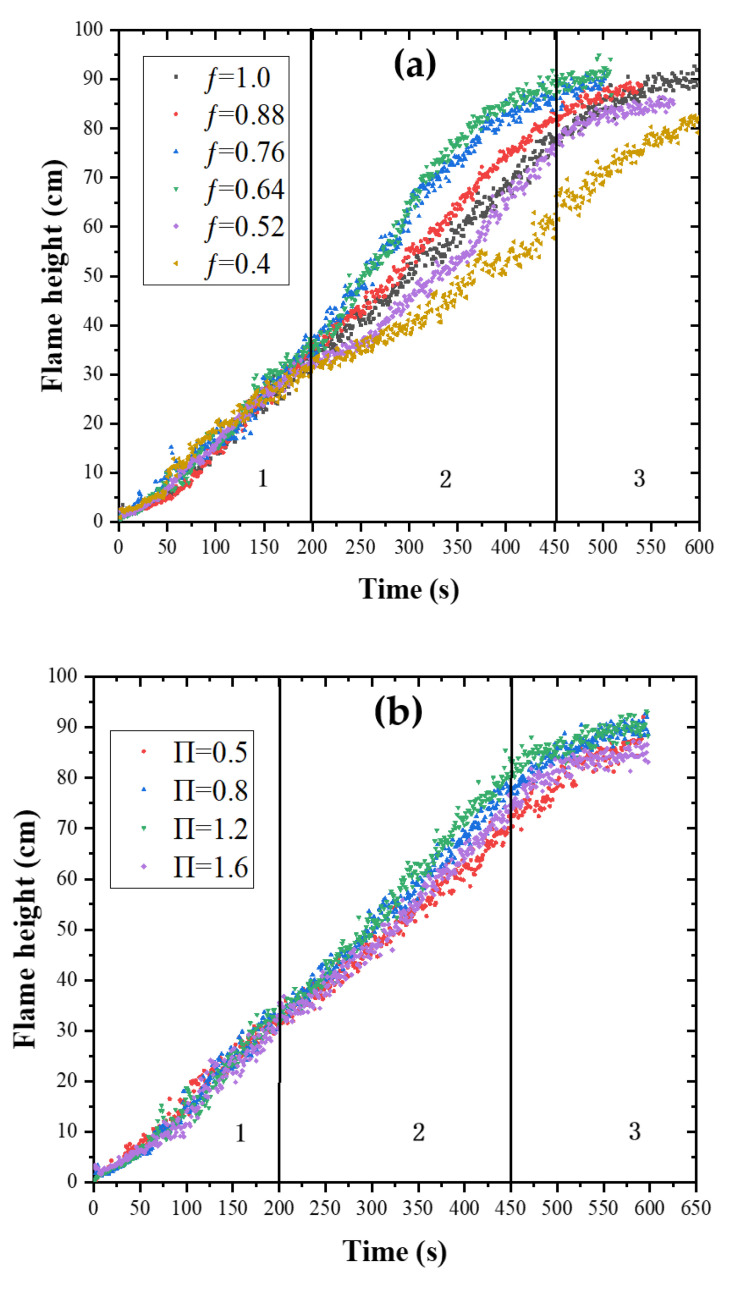
Flame height versus time under different experimental conditions: (**a**) П = 0.8, flame height under different fuel coverages; (**b**) ƒ = 1, flame height under different structure factors.

Flame height is an important parameter for fire risk analysis, and is defined as the vertical distance from the bottom of PMMA flame to flame top in this paper [40,41]. The flame height values are obtained through processing flame videos [28]. The processing method is explained as follows. Firstly, the videos are converted into color images, and then the color images are converted into gray images. Further, the brightness value of each pixel in the gray image is calculated. It is necessary to propose a threshold brightness value to distinguish the flame zone and non-flame zone. In this work, the threshold value was set at 230. The zone with the brightness value higher than 230 is deemed as the flame zone, and the length of the flame zone is the flame height. This image processing method and the threshold value were also used in previous study [42]. In order to analyze the influences of fuel coverage on PMMA flame height, the structure factor is fixed at 0.8, and the change curve of flame height with time under different fuel coverages are shown in Figure 5a. The changing trends of flame height versus time under different structure factors when the fuel coverage is 1 are shown in Figure 5b. 

From Figure 5a, it can be found that the flame height variation under different conditions can be divided into three stages: initial flame spread stage, flame spread development stage and stable flame spread stage. Time periods corresponding to the three stages are different under different conditions. During initial flame spread stage, the fluctuation of flame height is observed, which could be attributed to the existence of non-combustible air gap. The flame adherence occurs in the air gap, and thus the flame front cannot spread smoothly through the air gap, resulting in the flame height fluctuation. The changing trends of flame height with different fuel coverages are basically the same, and flame height tends to grow linearly along with time. In the flame spread development stage, flame height growth changes significantly due to the impact of structure factor and fuel coverage under different experimental conditions. During the stable flame spread stage, the PMMA surface has burnt completely and the flame height grows at a reducing rate and tends to be stable.

**Figure 6:**
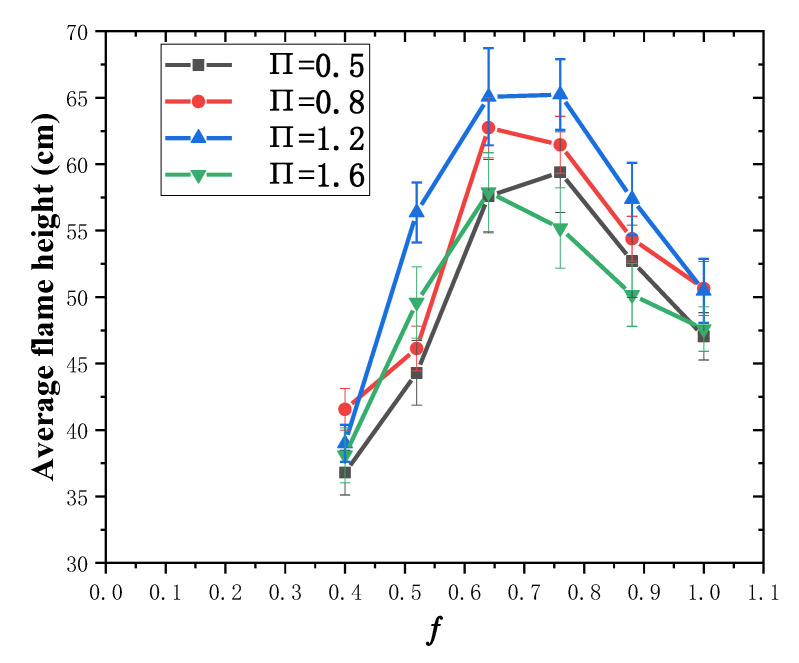
Average flame height under different experimental conditions (200 s–400 s).

In order to further compare flame heights under different structure factor and fuel coverage in flame spread development stage, this paper calculated the average flame height within 200 s–400 s, and the results are shown in Figure 6. It can be found that in the flame spread development stage, the average flame height first increases and then decreases as fuel coverage increases when the structure factor is fixed. It reaches the maximum when ƒ = 0.64–0.76, which is defined as the critical fuel coverage. Miller [6] tested different fuel coverage for upward flame spread over discrete PMMA, compared the flame height in the same time period, and also found that the flame height reached the maximum value at ƒ = 0.76. Similar conclusion is also drawn by Meng et al. [43], who used XPS as the experimental material and the structure factor was fixed at 0.2. They found that the average flame height first increases and then decreases with the decrease of porosity, which is negatively correlated with the fuel coverage. The reason for the above phenomena is explained as follows. When ƒ is smaller than the critical value, with the increase of the fuel coverage, more combustible pyrolysis gas is released and rises higher, leading to an increase in flame height. When ƒ is larger than the critical value, with the increase of fuel coverage, less air is entrained into the air gap, and thus inadequate combustion of PMMA plates occurs and the flame height is reduced. When the fuel coverage is fixed, the average flame height increases first and then decreases as the structure factor increases, and reaches the maximum when П = 1.2. As the structure factor increases, the chimney effect inside the vertical channel is enhanced, heat loss decreases and updraft speeds up to promote flame height to increase. In addition, as the structure factor increases, the front air supply is restricted more significantly and combustion efficiency is reduced as the width of sidewalls increases, inhibiting the increase in flame height. The competition of above two factors leading to the above phenomenon. 

Zhao [44] proposed a formula to predict the flame height:

(1) $$H_{f}=1.576\times 10^{7}{\left(\frac{V_{f}^{2}}{p}\right)}^{2/3}$$

where *V_f_* donates the flame spread rate,$\;H_{f}$ is the flame height and *p* is ambient pressure. Since the ambient pressure remains constant in this experiment, Equation (1) can be transformed to Equation (2).

(2) $$H_{f}\propto V_{f}^{4/3}$$

Using Equation (2), it is predicted that the changing trend of the flame spread rate is the same to that of the flame height, which is consistent with the experimental results.

### 3.3. Pyrolysis Front Propagation

**Figure 7:**
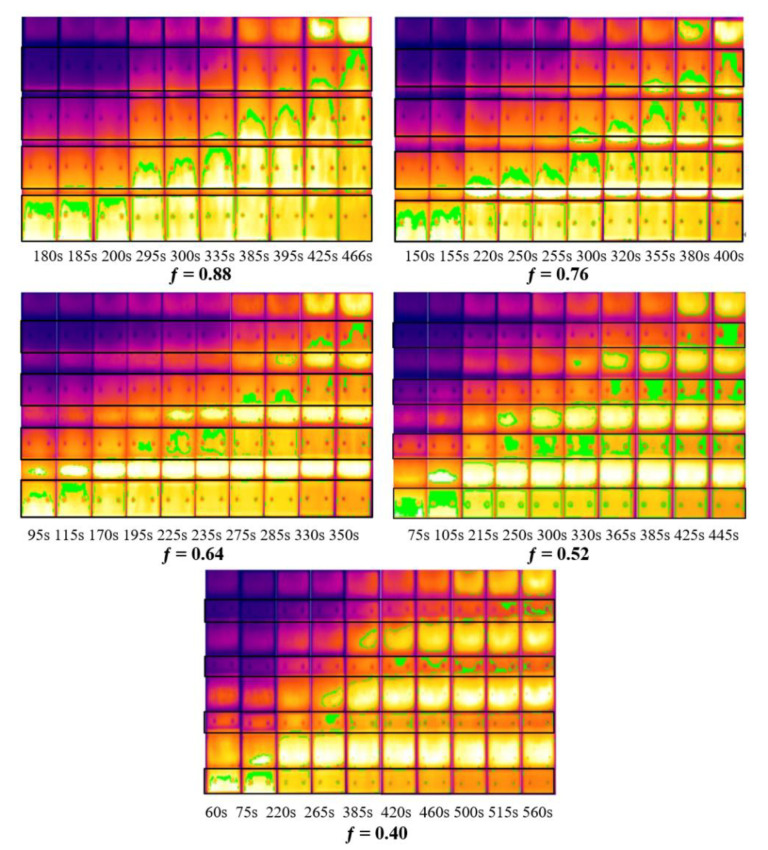
Change of pyrolysis front with time under different fuel coverages (П = 0.8).

In the experiment, the whole process of the upward flame spread over PMMA was recorded using the infrared camera, and the emissivity of PMMA was set at 0.92 [45]. The infrared video of PMMA surface was processed with ThermoX software to obtain data concerning the changes of PMMA pyrolysis front along with time. The pyrolysis temperature of PMMA is about 350 °C, which is marked in green in the infrared video. The IR pictures selected at different time under different fuel coverages for П = 0.8 are shown in Figure 7. The green part on the PMMA surface is the pyrolysis area, and the top of the green part is the position of PMMA pyrolysis front. Although the linear ignitor is used to ignite the bottom of PMMA, the pyrolysis front is not horizontal after the flame spreads over a certain distance, which could be attributed to the charring of PMMA, air entrainment from sample sides and the turbulent flame. This phenomenon is basically consistent with results obtained by Gollner et al. [46]. The heat flux is the highest in the center line of the vertical channel, which means the middle part of the PMMA sample receives more heat. Therefore, the pyrolysis front presents an inverted “V” shape, corresponding to the result of Comas et al. [47]. 

It is also found from Figure 7 that in the initial stage, the burn rate is relatively slow, resulting in a small change in the pyrolysis front over time. As the flame spreads upward, the increase in the height of the pyrolysis front presents an accelerated trend. The height of the pyrolysis front increases first and then decreases with the increase of fuel coverage, and reaches the maximum when the fuel coverage is 0.64. This is consistent with the results of Meng et al. [43].

The pyrolysis front spreads in three different patterns. The first pattern is that after the pyrolysis front on the surface of the first piece of PMMA reaches the top, it will jump over the air gap and appear at the bottom of the second piece of PMMA. The second pattern is that before the pyrolysis front on the surface of the first piece of PMMA spreads to the top, the pyrolysis phenomenon has appeared at the bottom of the second piece of PMMA. The third pattern is that after the surface of the first PMMA is pyrolyzed completely, pyrolysis front appears at the upper middle part of the second piece of PMMA, and then spreads up and down at the same time. In the concurrent flame spread over discrete thin papers, Park [26] also found the phenomenon of flame jumping. When the fuel coverage is 0.88 and 0.76, the second pattern of PMMA flame spread is observed. 

When the fuel coverage is 0.64, three different patterns of PMMA flame spread are all observed. When the flame front spreads from the first to the second piece of PMMA plate, the third pattern of flame spread is observed, as there is a long air gap among PMMA plates, the flame is in wave form and the middle and upper part of the second PMMA board is closer to the flame. Therefore, more flame heat flux is received. When the pyrolysis front spreads to the third piece of PMMA plate, the first pattern of flame spread is observed. When it spreads to the fourth piece of PMMA plate, the second pattern of flame spread is observed. The appearance of the different spread patterns is mainly caused by the unstable flame form and heat flow distribution. When the fuel coverage is 0.52 and 0.40, the third pattern of flame spread is observed. 

### 3.4. Theoretical Model of Flame Spread Rate

Flame spread rate is defined as the spread rate of the pyrolysis front [5,14,28,40,41]. The flame spread rate may be influenced with chemical structure of materials. Effects of initial molecular weight and thermal stability of PMMA plate on horizontal flame spreading behavior were studied by Kashiwagi et al. [48] The results indicated that the flame spread rate of the higher molecular weight PMMA sample was about four times larger than that of the low molecular weight sample. The sample with low initial molecular weight formed molten polymer which significantly affected flame spreading behavior and its rate. However, the effects of chemical structure of PMMA are not considered in this work since the fuel coverage and structure factor mainly influence the flow field and heat transfer rather than the chemical structure. 

**Figure 8:**
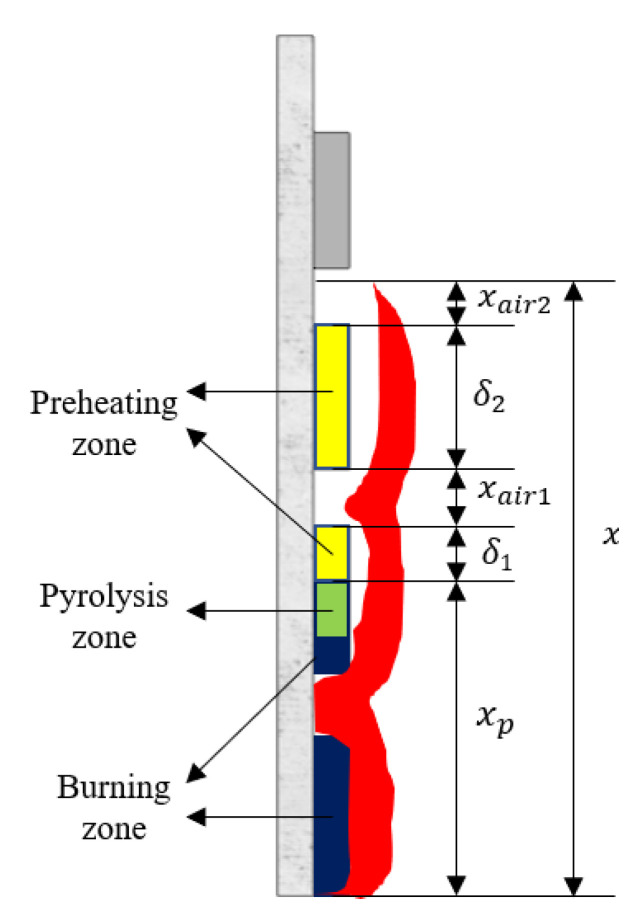
Physical model of the upward flame spread over discrete PMMA plate.

As shown in Figure 8, the physical model of flame spread over discrete PMMA was established according to the flame spread phenomenon observed in the experiment and the flame spread model established by previous researchers for continuous materials. Comparing to continuous flame spread, the air at the interval is heated and forms a negative pressure, making the flame close to the back wall. Therefore, the flame is observed in a wavy from. For this special flame shape, the heat flux received by each plate of PMMA at different heights depends on the vertical distance from the PMMA surface to flame.

For vertical continuous flame spread, the preheating zone length is the flame height subtracting the length of pyrolysis-zone. However, for discrete flame spread, flame coverage scope contains PMMA and an air gap. Therefore, this paper defines an effective preheating zone length as the flame height subtracting pyrolysis-zone length ($x_{p}$) and total length of air gaps ($x_{air}$). Its calculation formula is shown as Equation (3).

(3) $$\delta =x-x_{p}-x_{air}$$

The effective preheating zone length (δ) is the sum of the preheated PMMA length ($\delta_{1}$, $\delta_{2}$) between the flame front and pyrolysis front, as shown in Figure 8 and Equation (4).

(4) $$\delta =\delta_{1}+\delta_{2}$$

The total length of air gaps ($x_{air}$) is the sum of the air gaps length ($x_{air1}$, $x_{air2}$) between the flame front and pyrolysis front, as shown in Figure 8 and Equation (5).

(5) $$x_{air}=x_{air1}+x_{air2}$$

Based on the flame spread rate model of continuous solid proposed by Quintiere [49], this paper establishes the prediction model of discrete flame spread rate, supposing that the flame heat flux (${\dot{q}}_{f}^{\prime \prime }$) received by PMMA surface within the preheating zone and the flame spread rate are constant.

Quintiere [49] proposed a formula concerning flame spread rate and the preheating zone length, as shown in Equation (6). The flame spread rate ($V_{f}$) is proportional to the preheating zone length and inversely proportional to the ignition time ($t_{ig}$), since a shorter ignition time corresponds to faster forward moving of flame front.

(6) $$V_{f}=\frac{dx_{p}}{dt}=\frac{x-x_{p}}{t_{ig}}=\frac{\delta_{l}}{t_{ig}}$$

Substituting the effective preheating length (δ) for $\delta_{l}$ in Equation (6), Equation (7) could be obtained. Considering the effective preheating zone length and ignoring the existence of air gaps, the fuel spread rate ($V_{p,fuel}$) of PMMA surface is also determined by effective preheating zone length (δ) and ignition time ($t_{ig}$).

(7) $$V_{p,fuel}=\frac{d(x_{p}-x_{air})}{dt}=\frac{\delta }{t_{ig}}=\frac{x-x_{p}-x_{air}}{t_{ig}}$$

Miller et al. [6] found that the discrete flame spread rate ($V_{p}$) is negatively related with the fuel coverage (*f*). Conducting a series of experiments using different fuel coverages, they proposed an empirical formula concerning discrete flame spread rate, fuel spread rate (i.e., advancement rate of fuel pyrolysis zone) and fuel coverage, as shown in Equation (8).

(8) $$V_{p}=\frac{dx_{p}}{dt}=\frac{V_{p,fuel}}{f}$$

Substituting Equation (8) into Equation (7), Equation (9) could be obtained.

(9) $$V_{p}=\frac{\delta }{ft_{ig}}$$

The ignition time ($t_{ig}$) under constant external heat flux is defined as the heating time used for solid surface to be heated up to the ignition temperature, i.e., the time used for flame front to get pyrolyzed. In this paper, five points are uniformly selected on the surface of PMMA plate under different experimental conditions. The time used for each point to get pyrolyzed is recorded, and its average value is calculated as the average ignition time. 

In addition, the correlation between heat release rate and flame spread rate is also investigated in this work. Jiang et al. [19] proposed a formular concerning the dimensionless heat release rate ($Q^{*}$) and the dimensionless flame heigh for vertical PMMA fire:

(10) $$\frac{H_{f}}{L_{c}}\propto Q^{*0.58}$$

where $H_{f}$ is the flame height and $L_{c}$ is the characteristic length. In combination with Equations (2) and (10), a formular concerning the dimensionless heat release rate and flame spread rates is deduced:

(11) $$V_{f}\propto Q^{*0.435}$$

From Equation (11), it is deduced that the flame spread rate is positively correlated with the dimensionless heat release rate.

### 3.5. Experimental Flame Spread Rate and Comparison with Prediction

**Figure 9:**
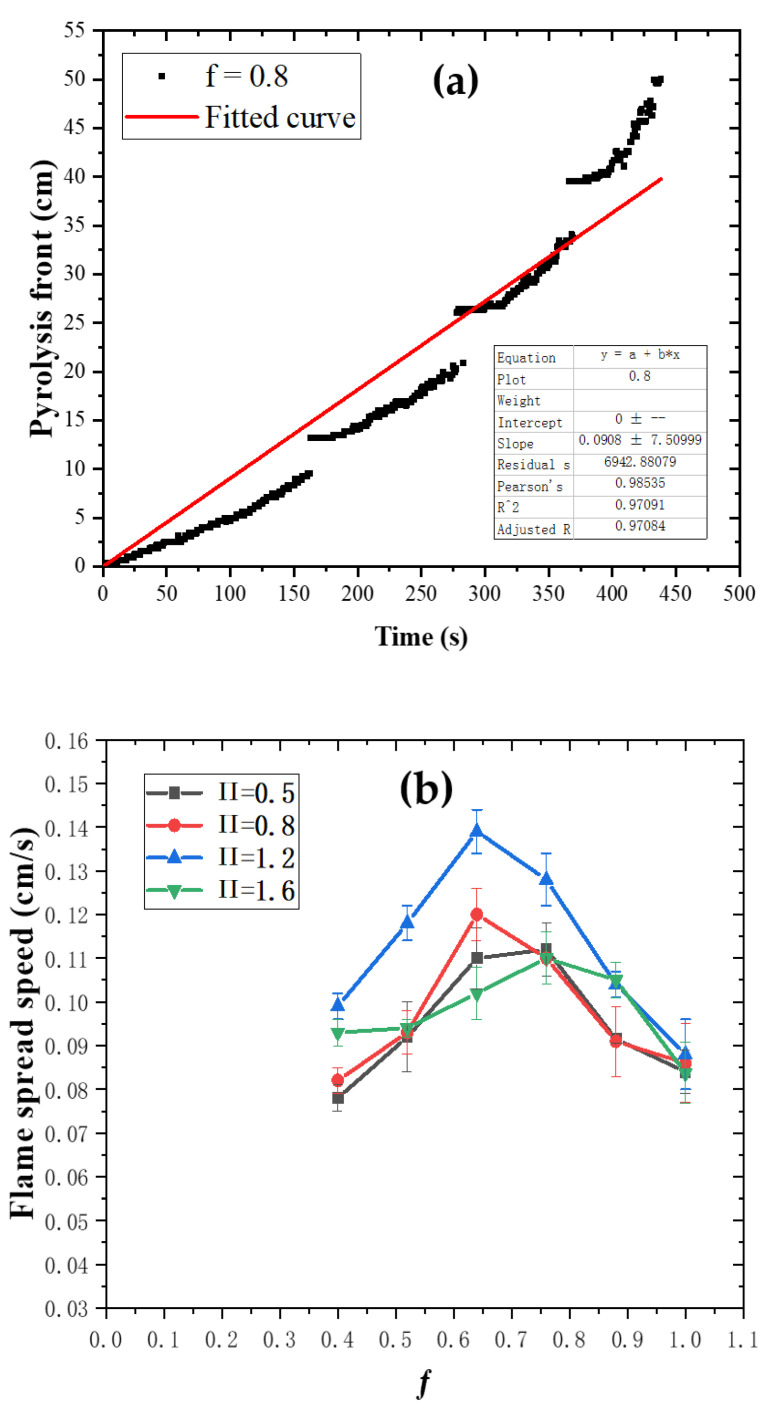
(**a**) Pyrolysis front position versus time and its fitting curve (П = 0.8, ƒ = 0.88); (**b**) change of experimental flame spread rate with structure factors and fuel coverage.

The typical change curve of the pyrolysis front position versus time is shown in Figure 9a. Linear fitting of Figure 9a is conducted, and the slope of the fitting line is the experimental flame spread rate [5,14,28,40,41]. The experimental flame spread rates under different structure factors and fuel coverages are shown in Figure 9b. 

When the structure factor is fixed, flame spread rate first increases and then decreases as fuel coverage increases, and reaches the maximum when ƒ = 0.64–0.76. Miller et al. [6] investigated the upward fire spread characteristics of discrete PMMA separated with non-combustible blocks and found that with the decrease of fuel coverage rate, the fire spread rate first increased and then decreased, which is consistent with the conclusion of this paper. For different fuel coverages, the flame spread rate is mainly affected by two factors. On the one hand, the distance between flame and PMMA surface is reduced as fuel coverage decreases. As a result, the flame gets closer to PMMA surface, which receives more flame heat flux, promoting the flame spread. Moreover, An et al. [50] proposed the following formulars for thermally thick thermoplastic materials.

(12) $$V_{f}\propto q_{conv}^{"}+q_{rw}^{"}$$

(13) $$q_{conv}^{"}\propto L_{c}^{-1/4}$$

where $q_{conv}^{"}$ and $q_{rw}^{"}$ denote convective and radiative heat flux, respectively. *L_c_* is characteristic length of experimental sample. As the fuel coverage decreases, the characteristic length of PMMA decreases, and thus the flame spread rate increases according to Equations (12) and (13). On the other hand, less PMMA burns inside the concave channel as the fuel coverage decreases. Therefore, heat release rate is reduced and longer time is used for flame height to reach the upper PMMA plate due to larger spacing, reducing the flame spread rate. The competition between the above two effects leads to the nonlinear variation of the flame spread rate with the fuel coverage. 

When the fuel coverage remains unchanged, the flame spread rate increases first and then decreases as the structure factor increases, and reaches the maximum when П = 1.2. As the structure factor rises, flame spread rate is mainly affected by two aspects. On the one hand, as the structure factor increases, the chimney effect enhances, heat loss decreases and updraft speeds up. All these factors will promote the increase of flame spread rate. On the other hand, air supply is restricted more significantly and combustion efficiency is reduced as the width of side walls increases, inhibiting the increase of flame spread rate. The coupling effects of the above two aspects causes the flame spread rate to increase first and then decrease with the increase in the structure factor.

**Figure 10:**
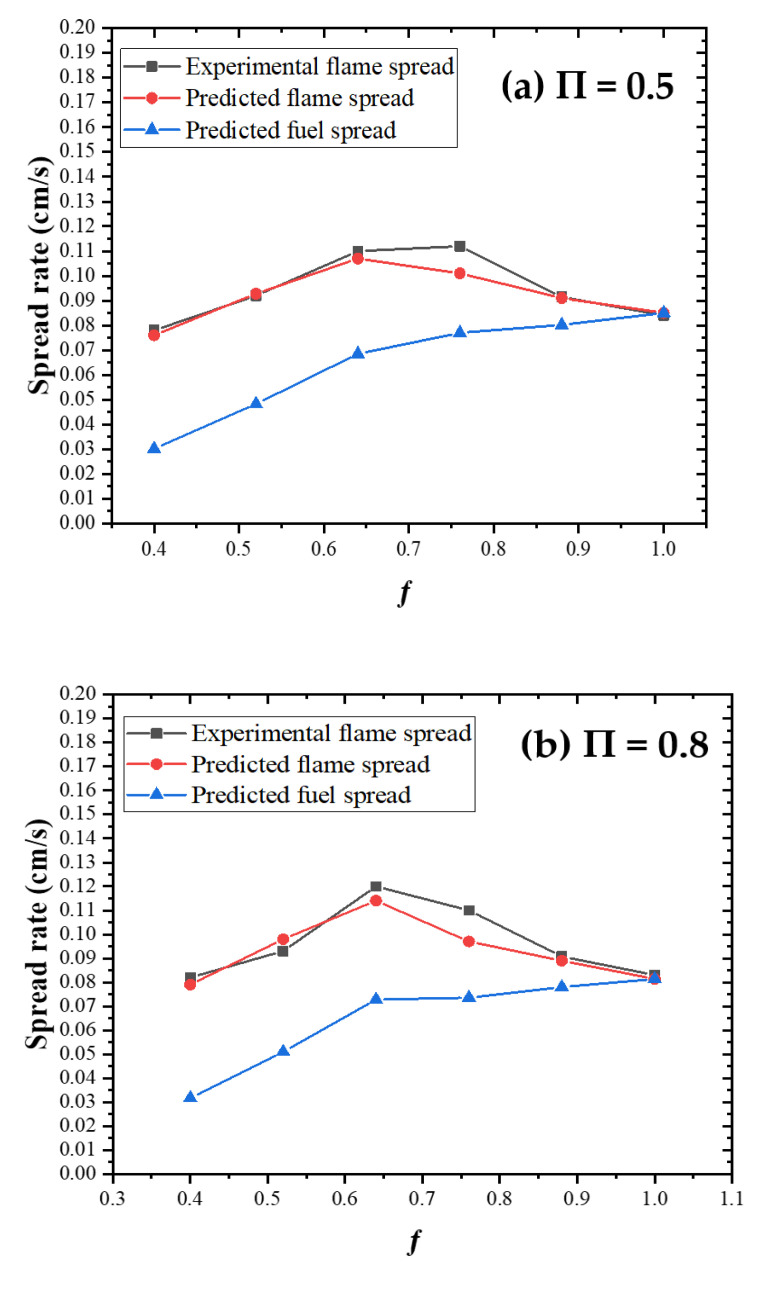

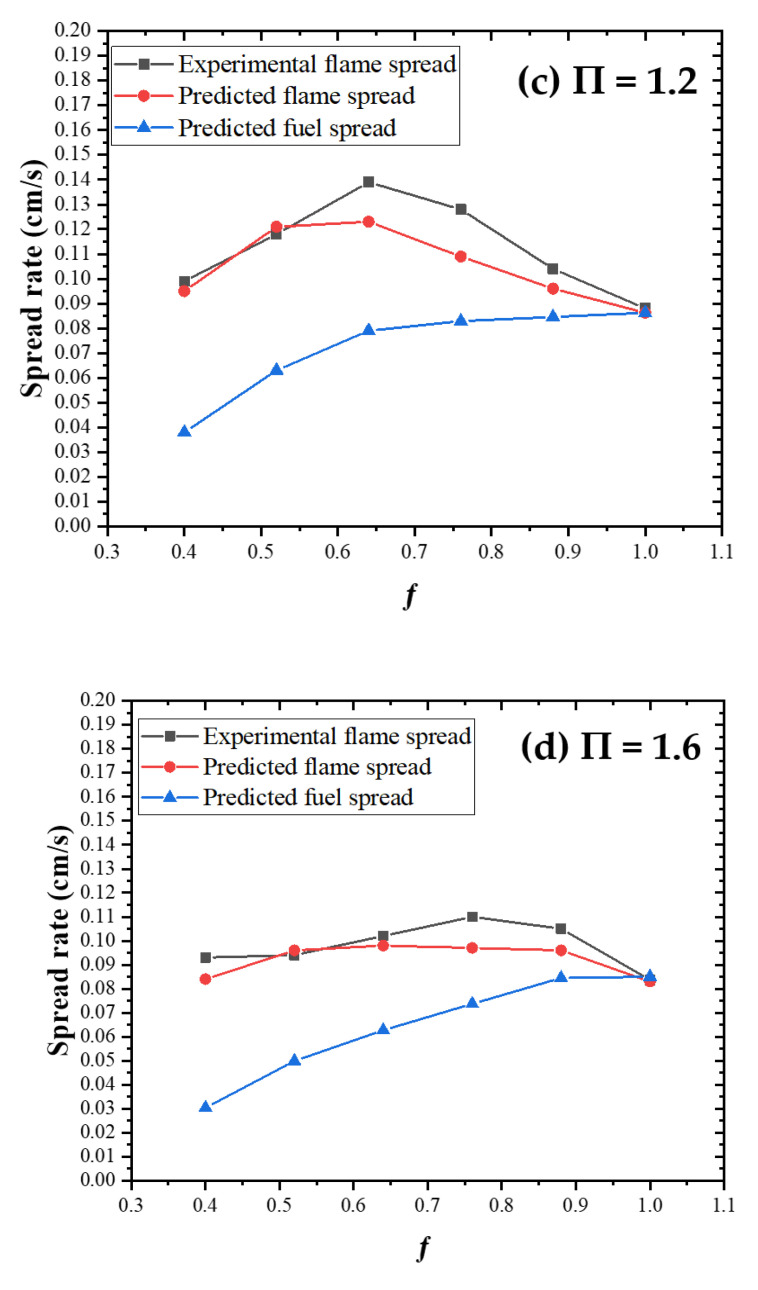
Predicted fuel spread rate, predicted flame spread rate and experimental flame spread rate under different conditions: (**a**) П = 0.5, (**b**) П = 0.8, (**c**) П = 1.2 and (**d**) П = 1.6.

By substituting the average ignition time and average length of effective preheating zones into Equations (7) and (9), the predicted fuel spread rate and predicted flame spread rate are calculated. The values of the predicted fuel spread rate, predicted flame spread rate and experimental flame spread rate are shown in Figure 10.

It can be found that the predicted flame spread rate obtained through Equation (9) is basically the same as the experimental flame spread rate, both of which tend to change in the same way along with fuel coverage. $V_{f,fuel}$ of PMMA increases as fuel coverage rises, which is basically consistent with the changing trend obtained by Miller [6].

## 4. Conclusions

In this paper, a series of experiments were carried out to study thermodynamics and kinetics characteristics of combustion of discrete polymethyl methacrylate plate with different spacings in concave structure. The conclusions were deduced from small-scale experiments, which will be validated in a future study using large-scale experiments. The conclusions are presented as follows.

(1) The flame shapes and flame height are significantly different under different concave structure factor and fuel coverage. The flame height could be divided into three stages: initial flame spread stage, flame spread development stage, stable flame spread stage. In the flame spread development stage, when the structure factor is fixed, the average flame height increases first and then decreases with the increase of fuel coverage, and reaches the maximum value when the fuel coverage is between 0.64 and 0.76.
(2) Three different patterns of pyrolysis front propagation are observed for different fuel coverages due to the existence of air gaps. When the structure factor is fixed, the flame spread rate first increases and then decreases with an increase in fuel coverage, and reaches the maximum value when the fuel coverage is between 0.64 and 0.76. When the fuel coverage is fixed, the flame spread rate first increases and then decreases with increasing structure factor, and reaches the maximum value when the structure factor is 1.2.
(3) A model for predicting the flame spread rate of discrete polymethyl methacrylate plates is established based on the model suitable for continuous flame spread. The predicted flame spread rate is consistent with the experimental results. It is found that the fuel spread rate of discrete polymethyl methacrylate plates rises with the increase of fuel coverage.

This study contributes basic data and theory for fire safety science of polymethyl methacrylate. Moreover, models established and results obtained in this work are beneficial to thermal hazard evaluation and fire safety design of buildings employing polymethyl methacrylate. 

## Figures and Tables

**Table 1:** Parameters of thermophysical properties of polymethyl methacrylate (PMMA) [36].

Material	$k/Wm^{-1}K^{-1}$	$\rho /Kg\;m^{-3}$	$c_{p}/J\;Kg^{-1}K^{-1}$	$T_{p}/K$	$T_{f}/K$	$\sigma /\;W^{2}m^{-4}K^{4}$
PMMA	0.05	1190	1400	623	1073	5.67 × $10^{-8}$

**Table 2:** Experimental conditions concerning concave structure factor.

Experimental Condition Number	1#	2#	3#	4#
Back wall width/cm	10	10	10	10
Side wall width/cm	5	8	12	16
Structure factor (П)	0.5	0.8	1.2	1.6

## Data Availability

The data presented in this study are available in article.

## Nomenclature

$V_{f}$	flame spread rate [cm/s]
$x_{p}$	vertical length of pyrolysis zone [cm]
П	structure factor (the ratio of the side wall width to back wall width)
x	flame height (cm)
δ	effective preheating zone length (preheating zone length subtracting vertical length of air gaps) (cm)
$V_{p}$	flame spread rate (advancement rate of total pyrolysis zone) (cm/s)
$V_{p,fuel}$	fuel spread rate (advancement rate of fuel pyrolysis zone) (cm/s)
$x_{air}$	total length of air gaps in preheating zone (cm)
ƒ	fuel coverage
$\delta_{l}$	preheating zone length (cm)
$t_{ig}$	ignition time (s)
*k*	thermal conductivity $({\mathrm{Wm}}^{-1}K^{-1}$)
*ρ*	density $({\mathrm{kg}\;m}^{-3}$)
$c_{p}$	specific heat $({J\;\mathrm{Kg}}^{-1}K^{-1}$)
$T_{p}$	pyrolysis temperature (K)
σ	Stefan-Boltzmann constant $(W^{2}m^{-4}K^{4}$)
$T_{f}$	flame temperature (K)
$Q^{*}$	dimensionless heat release rate
*L_c_*	characteristic length (cm)
$q_{conv}^{"}$	convective heat flux $({W\;m}^{-2}$)
$q_{rw}^{"}$	radiative heat flux $({W\;m}^{-2}$)
**Abbreviations**	
PMMA	polymethyl methacrylate
